# The density of tumour infiltrating lymphocytes in oesophago-gastric cancer varies with disease stage, geographical region and treatment: a post hoc analysis of nine phase III clinical trials

**DOI:** 10.1007/s10120-026-01739-6

**Published:** 2026-05-05

**Authors:** Georgina A. Keogh, Nina Šefčovičová, Tomio Arai, Myeong-Cherl Kook, Jon P. Laye, William H. Allum, Sameira Arif, Avani Athauda, Hee Kyung Chang, Jae-Ho Cheong, Mee-Yon Cho, David Cunningham, Lara Heij, Andrew F. Irvine, Hee Sung Kim, Hyunki Kim, Young-Woo Kim, Ruth E. Langley, Sung Hak Lee, Katharina von Loga, Matthew G. Nankivell, Takashi Oshima, Russell D. Petty, Xiuxiang Tan, Shiro Tanaka, Akira Tsuburaya, Judith de Vos-Geelen, Nicholas P. West, Jake Emmerson, Jamie R. Stokes, Derek R. Magee, David A. Cairns, Heike I. Grabsch

**Affiliations:** 1 Gastrointestinal and Lymphoma Unit, Royal Marsden NHS Foundation Trust, London, UK; 2 Department of Pathology, GROW - Research Institute for Oncology and Reproduction, Maastricht University Medical Center+, Maastricht, The Netherlands; 3 Department of Pathology, Tokyo Metropolitan Institute for Geriatrics and Gerontology, Tokyo, Japan; 4 Department of Pathology, Center for Gastric Cancer, National Cancer Center, Goyang, Republic of Korea; 5 Division of Pathology and Data Analytics, Leeds Institute of Medical Research at St James’s, University of Leeds, Leeds, UK; 6 Department of Surgery, Royal Marsden NHS Foundation Trust, London, UK; 7 Department of Pathology, Kosin University Gospel Hospital, Busan, Republic of Korea; 8 Department of Surgery, Yonsei University College of Medicine, Seoul, Republic of Korea; 9 Department of Pathology, Wonju Severance Christian Hospital, Wonju-si, Gangwon-do Republic of Korea; 10 Department of General, Visceral and Transplantation Surgery, University Hospital Essen, Essen, Germany; 11 Institute of Pathology, University Hospital Essen, Essen, Germany; 12 Department of Medicine 2 (Medical Faculty), RWTH Aachen University, Aachen, Germany; 13 Department of Pathology and Bioinformatics, Erasmus University Medical Center, Rotterdam, The Netherlands; 14 Pathology Department, Mid Yorkshire Hospitals NHS Trust, Wakefield, UK; 15 Department of Pathology, Chung-Ang University Hospital, Seoul, Republic of Korea; 16 Department of Pathology, Yonsei University College of Medicine, Seoul, Republic of Korea; 17 Department of Public Health & AI, National Cancer Center Graduate School of Cancer Science and Policy, and Center for Gastric Cancer and Department of Surgery, National Cancer Center, Goyang, Republic of Korea; 18 UCL Innovative Clinical Trials Unit, University College London, London, UK; 19 Department of Hospital Pathology, College of Medicine, Seoul St. Mary’s Hospital, The Catholic University of Korea, Seoul, Republic of Korea; 20 Waiv, Paris, France; 21 Department of Gastrointestinal Surgery, Kanagawa Cancer Center, Yokohama, Japan; 22 Division of Cancer Research, School of Medicine, University of Dundee, Dundee, UK; 23 Department of General Surgery, Pancreatic Disease Center, Ruijin Hospital, Shanghai Jiao Tong University School of Medicine, Shanghai, China; 24 Research Institute of Pancreatic Diseases, Shanghai Key Laboratory of Translational Research for Pancreatic Neoplasms, Shanghai Jiao Tong University School of Medicine, Shanghai, China; 25 Center for Clinical and Translational Research, Kyushu University Hospital, Kyushu University, Fukuoka, Japan; 26 Department of Surgery, Fukuzawa Clinic, Yokohama, Japan; 27 Department of Internal Medicine, Division of Medical Oncology, GROW - Research Institute for Oncology and Reproduction, Maastricht University Medical Center+, Maastricht, The Netherlands; 28 Department of Histopathology, Leeds Teaching Hospitals NHS Trust, Leeds, UK; 29 Clinical Trials Research Unit, Leeds Institute of Clinical Trials Research, University of Leeds, Leeds, UK; 30 School of Computer Science, University of Leeds, Leeds, UK; 31 HeteroGenius Limited, Leeds, UK

**Keywords:** Tumour infiltrating lymphocytes, Oesophago-gastric cancer, Age, Sex, Disease stage, Treatment

## Abstract

**Background:**

Tumour infiltrating lymphocytes (TILs) are a key component of the tumour microenvironment. To establish a clinically relevant TILs cut-off for patients with oesophago-gastric (OG) cancer, it is essential to know whether TILs density varies by patient and/or disease characteristics.

**Materials and methods:**

TILs were quantified as TILs/mm^2^ (TILs density) by a deep-learning algorithm applied to digitised Haematoxylin/Eosin (H&E)-stained biopsies and resection specimens from 4628 patients from nine phase III trials. 4533 patients with TILs density and matched clinicopathological data were included in the final analyses. Associations between TILs density, disease stage, geographical region (UK versus Asia), sex, age, and treatment were analysed.

**Results:**

Median TILs density was higher in pre-treatment biopsies from patients with early-stage versus late-stage disease (962 vs 479 TILs/mm^2^, *p* < 0.001). Within the same geographical region and disease stage, TILs density was similar across different chemotherapy regimens. In UK-led trials of early-stage disease, post-chemotherapy resections showed higher TILs density than chemotherapy-naïve resections (618 vs 571 TILs/mm^2^, *p* = 0.003). TILs density was higher in Asian tumours compared to UK tumours (1419 vs 571 TILs/mm^2^, *p* < 0.001). No significant associations were observed with age or sex.

**Conclusions:**

This is the largest study to date evaluating TILs density in OG cancer. TILs density varied with stage and geographical region but not by age or sex. These findings may explain enhanced response to immunotherapy observed in published studies of patients with early-stage disease and highlight the need to account for baseline TILs heterogeneity when interpreting TILs as a possible biomarker in future studies.

**Supplementary Information:**

The online version contains supplementary material available at 10.1007/s10120-026-01739-6.

## Highlights

- Tumour infiltrating lymphocyte (TILs) density in oesophago-gastric cancer varies by disease stage and geographical region.
- TILs density does not vary by age or sex.
- TILs density increases after chemotherapy.
- TILs density is similar across different regimens after chemotherapy.

**Supplementary Information:**

The online version contains supplementary material available at 10.1007/s10120-026-01739-6.

## Introduction

Oesophageal and gastric (OG) cancer remain a major global health burden ranking as sixth and fourth leading causes of cancer-related deaths worldwide in 2021, respectively [1]. Incidence rates vary geographically with the highest rates observed in Asia [1] largely due to differences in genetic predisposition, lifestyles factors (notably smoking and alcohol consumption), and *Helicobacter pylori* infection [2].

Treatment strategies for OG cancer patients are similar but vary depending on disease stage and histopathological subtype. For patients with early-stage, locally advanced, resectable adenocarcinoma, standard patient management includes a combination of chemotherapy and surgery [3–6]. In patients with late-stage, unresectable, locally recurrent or metastatic disease, palliative chemotherapy remains the mainstay of treatment with the potential addition of targeted therapy, such as HER2-directed agents, or immune checkpoint inhibitors depending on tumour biomarker status [7–10].

Despite these therapeutic advancements, survival remains poor, and treatment selection is still primarily based on disease stage, performance status and patient preference [11, 12]. While specific molecular biomarkers may guide the use of targeted therapy in patients with late-stage disease [8, 13], there are no validated biomarkers to predict response to standard cytotoxic chemotherapy. Notably, patients with similar disease stage and performance status can experience markedly different response to the same chemotherapeutic agents [3, 14, 15]. This highlights the unmet clinical need for reliable predictive biomarkers that could guide treatment decisions in OG cancer patients irrespective of disease stage, age, sex or ethnicity.

Primary tumour infiltrating lymphocytes (TILs) are a key component of the tumour microenvironment [16, 17]. Manual Haematoxylin/Eosin (H&E)-based stroma TILs scoring is included in the routine pathological assessment in breast cancer since 2018 [17]. Several studies investigated TILs density and prognosis in OG cancer [18–21], however, few have considered demographic factors such as age, sex and ethnicity, which shape host immunity.

Immune function typically declines with age [22], males tend to exhibit lower immune responses than females [23], and immune responses can vary significantly across different ethnic groups [24–26]. Evidence from other tumour types suggest that TILs decline with age [27–29], are often higher in females [30, 31], and vary with ethnicity, with higher levels reported in Asian patients across several cancers [32–35]. TILs density also tends to decrease with advancing disease stage [36]. However, current data on TILs and their relationship with demographic and clinicopathological factors in OG cancer patients is limited, inconsistent and mainly derived from retrospective single-centre cohorts [37, 38]. A comprehensive assessment of TILs density across diverse clinical populations is therefore needed to inform subsequent analyses of its prognostic and predictive relevance.

We hypothesised that TILs density in OG cancer varies according to demographic and clinicopathological factors being higher in younger patients, females, patients treated in Asia and patients with early-stage tumours.

The aim of this study was to characterise and compare TILs density across OG cancer patients stratified by disease stage, treatment modality and clinicopathological factors, using deep learning algorithm in digital H&E-stained tissue sections from 4628 patients enrolled in nine phase III OG cancer trials from the UK (OE02, OE05, ST02 (MAGIC), ST03, REAL3, COG, GO2), and Asia (SAMIT and CLASSIC.) This large global dataset provides a unique opportunity to define the landscape of TILs density in OG cancer and establish the foundation for future prognostic and predictive analyses.

## Methods

### Patient populations

#### UK-led trials in patients with early-stage oesophageal, junctional or gastric cancer

1. Medical Research Council (MRC) ST02 trial (also known as the MAGIC trial).

This phase III trial randomised patients with gastric or lower third oesophageal adenocarcinoma to either surgery alone or peri-operative epirubicin, cisplatin, 5-Fluorouracil (5-FU) (ECF) chemotherapy [39]. For the current TILs density analysis, digital H&E-stained slides were available from 305 resection specimens (62% of 494 the patients who had a resection). Pre-treatment biopsies were not available from this trial. (Further details see Table 1.)

**Table 1:** Basic characteristics of the clinical trials included in the current study

Disease type	UK-led							Asia-led	
	Locally advanced resectable (early stage disease)				Locally advanced irresectable, recurrent, or metastatic (late stage disease)			Locally advanced resectable (early stage disease)	
	ST02/MAGIC	OE02	OE05	ST03	REAL3	COG	GO-2	SAMIT	CLASSIC
Inclusion criteria	Stage II	Stage I-III	Stage I-III	Stages Ib-III	1st line advanced	2nd line advanced	Older, frail, 1st line advanced	pT4a-pT4b	Stage II—IIIb
Location	G, EGJ, lower O	EGJ, all O	EGJ, all O	G, EGJ	G, EGJ, all O	EGJ, all O	G, EGJ, all O	Stomach	Stomach, EGJ
Histology type	adenoca	SCC, adeno	adenoca	adenoca	adenoca	SCC, adeno	SCC, adeno	adenoca	adenoca
No. of trial pts	503	802	897	1063	553	449	559	1495	1035
Treatment regimen	Peri-operative ECF vs. S alone	Neoadjuvant CF vs. S alone	Neoad CF vs. ECX	Peri-operative ECX vs. ECX + bev + Bev maintenance	EOC/EOX vs. EOC/EOX + panitumumab	Gefitinib vs. Placebo	3 chemo intensity levels A, B,C CAPOX) OR Low level CAPOX vs. BSC	Adjuvant paclitaxel + UFT/S-1 vs. Monotherapy S-1/UFT	Adjuvant CAPOX vs S alone
Overall survival (OS)	5-year OS: 36.3% C + S vs 23.0% S	5-year OS: 23.0% C + S vs 17.1% S alone HR, 0.84	Median OS: 23.4 mo CF vs 26.1 mo ECX; HR 0.90	3-year OS: 50.3% ECX vs 48.1% ECX + bev; HR 1.09	Median OS: 8.8 mo EOC + P vs 11.3 mo EOC; HR 1.37	Median OS: 3·73 mo gefitinib vs 3·67 mo placebo; HR 0.09	Levels B vs A and C vs A had non-inferior PFS	3-year OS: 55.8% mono vs 59.3% sequential, HR 0.93 3-year OS: 54.3% UFT vs 60.7% S-1; HR 0·81	5-year OS: 78% Adj CAPOX vs 69% S alone; HR 0.66
Interpretation	Peri-op chemo improves OS	Neoad chemo improves OS	No difference between therapy regimens	No difference between therapy regimens	No difference between therapy regimens	Gefitinib does not improve OS	Frail, older population may benefit from lower chemo doses	No difference between therapy regimens	Improved OS for adj chemo

Abbreviations: G: gastric. EGJ: oesophago-gastric junction. O: oesophageal. Adenoca: adenocarcinoma. SCC: squamous cell carcinoma. No.: number. Pts: patients. Peri-op: peri-operative. ECF: eprubicin + cisplatin + 5-fluorouracil. S: surgery. C + S: chemotherapy and surgery. Neoad: neoadjuvant. CF: cisplatin + 5-fluorouracil. ECX: epirubicin + cisplatin + capecitabine. Bev: bevacizumab. EOC/EOX: epirubicin + oxaliplatin + capecitabine. CAPOX: capecitabine + oxaliplatin. BSC: best supportive care. UFT: tegafur + uracil. S-1: tegafur + gimeracil + oteracil. Adj: adjuvant. PFS: progression-free survival. HR: hazard ratio

2. MRC OE02 trial.

This phase III trial randomised patients with oesophageal or junctional cancer to either surgery alone or neoadjuvant chemotherapy (2 cycles cisplatin and 5-FU (CF)) followed by surgery [15]. For the current TILs density analysis, digital H&E-stained slides were available from 542 patients. Of these, 220 had paired biopsy and resection samples, 84 had pre-treatment biopsies only and 238 had resection specimens only. In total, biopsy material from 304 patients (38% of 802 randomised patients diagnosed by biopsy) and resection material from 458 (62% of 743 patients who had a resection) was investigated. (Further details see Table 1.)

3. MRC OE05 trial.

This phase III trial randomised patients with oesophageal or junctional adenocarcinoma to either 2 cycles CF (OE02 style) chemotherapy or 4 cycles epirubicin, cisplatin, and capecitabine (ECX) chemotherapy followed by surgery [40]. For the current TILs density analysis, digital H&E-stained slides were available from 826 patients. Of these, 557 had paired biopsy and resection samples, 201 had pre-treatment biopsies only and 84 had resection specimens only. In total, biopsy material from 758 patients (85% of 897 randomised patients) and resection material from 641 (85% of 751 patients who had a resection) was investigated. (Further details see Table 1.)

4. MRC ST03 trial.

This phase II/III trial randomised patients with gastric or lower third oesophageal adenocarcinoma to either peri-operative ECX or ECX plus bevacizumab [41]. For the current TILs density analysis, digital H&E-stained slides were available from 937 patients. Of these, 605 had paired biopsy and resection samples, 271 had pre-treatment biopsies only and 122 had resection specimens only. In total, biopsy material from 937 patients (88% of 1063 randomised patients) and resection material from 727 (81% of 895 patients who had a resection) was investigated. (Further details see Table 1.)

#### UK-led trials in patients with late-stage, locally advanced, recurrent or metastatic oesophageal, junctional or gastric cancer

1. REAL3 trial.

This phase III trial randomised patients with treatment-naïve OG adenocarcinomas to either up to 8 cycles of epirubicin, oxaliplatin and capecitabine (EOX) or to a modified-dose EOX plus panitumumab [42]. For the current TILs density analysis, digital H&E-stained slides were available from 282 pre-treatment primary tumour biopsies (51% of 553 randomised trial patients). Biopsies from metastatic sites and material from a previous resection were excluded from this study. (Further details see Table 1.)

2. COG trial.

This phase III trial randomised patients with oesophageal cancer or Siewert type I/II junctional cancer who had progressed after chemotherapy to either placebo or gefitinib [43] For the current TILs density analysis, digital H&E-stained slides were available from 300 pre-treatment biopsies (67% of 449 randomised trial patients). Biopsies from metastatic sites and material from a previous resection were excluded from this study. (Further details see Table 1.)

3. GO2 trial.

This phase III trial randomised elderly and/or frail patients with chemotherapy-naïve OG cancer to three different dose levels of oxaliplatin plus capecitabine chemotherapy [44]. For the current TILs density analysis, digital H&E-stained slides were available from 282 pre-treatment biopsies (50% of 559 randomised trial patients). Biopsies from metastatic sites and material from a previous resection were excluded from this study. (Further details see Table 1.)

#### Asia-led trials in patients with early-stage locally advanced resectable gastric cancer

1. Japanese SAMIT trial.

This phase III trial randomised patients with pT4a/b gastric adenocarcinoma after D2 gastrectomy to either adjuvant monotherapy with tegafur and uracil (UFT) only or monotherapy of S-1 only, or sequential therapy of paclitaxel followed by UFT or paclitaxel followed by S-1 [45]. For the current TILs density analysis, digital H&E-stained slides were available from 543 (36% of 1495 randomised trial patients) resection specimens. (Further details see Table 1.)

2. Korean CLASSIC trial.

This phase III trial randomised patients with stage II-IIB gastric adenocarcinoma after D2 gastrectomy to either 8 cycles of adjuvant capecitabine and oxaliplatin chemotherapy or to observation alone [46]. We previously analysed TILs density in digital H&E-stained slides from 549 CLASSIC trial resection specimens (53% of 1039 randomised trial patients) [19]. The previously published TILs density data were used in the current study for further analyses. (Further details see Table 1.)

### Measurement of tumour infiltrating lymphocytes (TILs)

H&E stained tissue sections of endoscopic biopsies and resection specimens from all trials except GO2 were scanned at 20 × or 40 × magnification using an Aperio Scanner (Leica Biosystems, UK). For GO2 trial patients, H&E-stained slides were scanned with Hamamatsu’s NanoZoom scanner (Hamamatsu Photonics, Japan). Images were uploaded to HeteroGenius-MIM image analysis software (HeteroGenius Ltd., Leeds, UK).

An International UGI TILs working group was established and several virtual meetings were held to develop a consensus on the methodology for TILs quantification. A protocol for region-of-interest identification and manual annotation procedures was agreed: in endoscopic biopsy samples, all tumour areas including areas with high grade dysplasia were individually outlined. Areas containing fibrin, erosion, mechanically damaged tissue, strips of neoplastic epithelial cells without stroma, extracellular mucin, and areas with low grade dysplasia were excluded from the annotation. For resection specimens, two 3 mm diameter circles (‘virtual tissue microarray cores’) were manually placed in the areas of highest tumour content avoiding lymphoid aggregates. This mirrors the methodology used in the CLASSIC trial TILs density study where physical 3 mm diameter tissue microarray cores from areas with highest tumour content were sampled for analysis [19].

The deep learning model we used in the CLASSIC trial study was updated for the current study to enable automatic background detection within the manually defined regions of interest for OE02, OE05, ST02 (MAGIC), ST03, COG and SAMIT trials *(for further details see *[47]*)*. Due to marked variability in the H&E-staining between GO2, REAL3 and the other trials, additional training of the existing algorithm was required to ensure accurate TILs detection in these datasets.

Extensive quality control of the automated TILs detection was performed manually at multiple stages by a senior pathologist (HIG) and senior technician (JL) both blinded to clinicopathological variables. Quality control was performed on randomly selected 10% of images as well as all on outliers defined as having values greater than 2 standard deviations of the mean. In these cases, the TILs detection overlay was compared with the H&E findings. In addition, the slides were processed through a tissue section quality software (HistoQC, an open-source quality control tool for digital pathology slides, see JCO Clin Cancer Inform 3, 1–7, 2019).

### Calculation of TILs density per patient

TILs density was calculated for both endoscopic biopsies and virtual cores from resection specimens using the same approach. The following values were extracted from the image analysis software for each annotated tumour containing region: (1) size of the region, (2) size of the automatically detected background/empty space in the region and (3) the number of TILs per region. To determine the true tumour tissue area in mm^2^, the automatically detected background was subtracted from the tumour region size. TILs density per patient was calculated by (A) adding up the number of TILs from the different regions (total number of TILs per patient), (B) adding up the true tumour region sizes (total tumour region per patient) and then division of (A) by (B) to obtain the final TILs density (TILs/mm^2^) per patient.

**Fig. 1:**
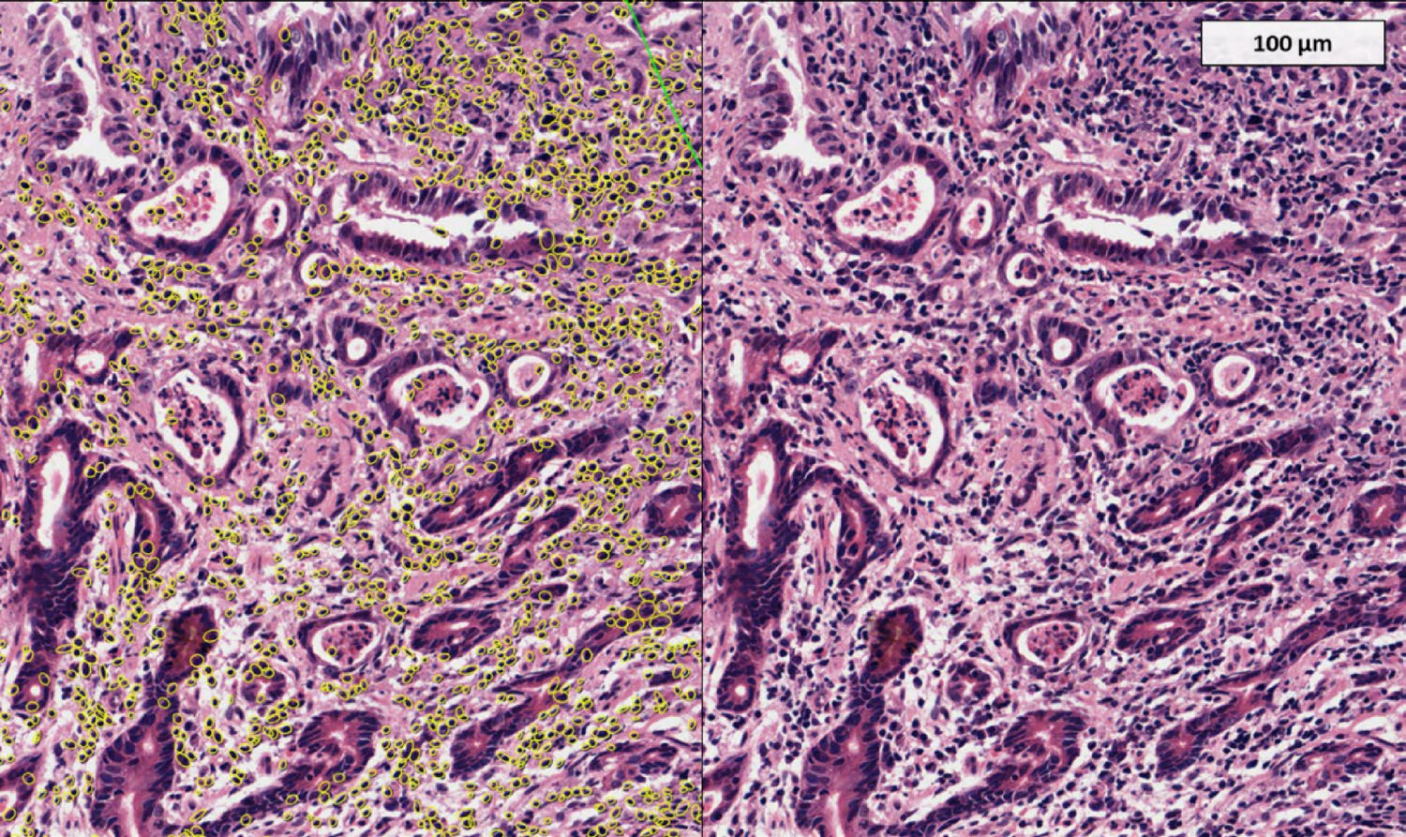
Illustration of automatic TILs detection. Left panel: TILs detected by the deep learning algorithm are highlighted by yellow outlines. These are present within the stroma and the adenocarcinoma itself. Green line in the top right hand corner outlining the region of interest. Right panel: Same H&E picture without TILs detection for reference

We did not distinguish between intraepithelial and stromal TILs in line with a previous study which suggested the TILs density per total tumour area as the best and most robust index for TILs density in gastric cancer [48]. An illustration of the TILs detection is provided in Fig. 1.

### DNA mismatch repair status

DNA mismatch repair (MMR)/microsatellite instability (MSI) data was available from previous studies for OEO2, MAGIC, CLASSIC and GO-2 [49–52]. However, as only one adenocarcinoma was found to be MMR deficient and MSI in OE02, the relationship between TILs density and MMR/MSI status was not investigated in OE02. The relationship between TILs density (continuous data) and MMR status was evaluated in MAGIC, CLASSIC and GO2. MMR data was not available for the rest of the trials.

### Ethics

This study was approved by the respective local institutional review boards and by the South East Research Ethics Committee, London, UK, REC reference: 07/H1102/111.

### Statistical analysis

Statistical analyses were performed using IBM SPSS statistics software (version 28). Descriptive statistics including the number of tumour regions per biopsy, total tumour area per sample type per patient, as well as mean, standard deviation, median, minimum, maximum, interquartile range and percentiles of the TILs density were calculated.

First, the median TILs density values were calculated for each treatment arm within each trial and compared across disease stage (early-stage vs late-stage disease), geographical region (UK-led vs Asia-led trials), specimen types (pre-treatment endoscopic biopsies, chemotherapy-naïve resection specimens, and post-chemotherapy resection specimens).

Second, the association between TILs density and clinicopathological variables per patient group was evaluated. For this, patients were grouped as follows:

Group 1. UK-led resections without chemotherapy (OE02, MAGIC).

Group 2. UK-led post-chemotherapy resections (OE02, OE05, ST03, MAGIC).

Group 3. Asia-led resections (SAMIT, CLASSIC).

Group 4. Pre-treatment biopsies from early-stage disease (OE02, OE05, ST03).

Group 5. Biopsies from late-stage disease (REAL3, COG).

GO2 trial patients were not included in the groups above and were analysed separately, because the TILs density was significantly different from the other late-stage disease trials.

The TILs density of all trial arms was added up per group and the mean and the percentiles per group were calculated.

In both stages of the analysis, we categorised the TILs density into low, medium and high using the percentiles as cut-offs. Low TILs density was defined as below 25th percentile, medium as between 25 and 75th percentile and high as above 75th percentile. To determine whether distinct clinicopathological features were associated with extreme immune phenotypes, we focused our analyses on comparing results from OG cancer patients in the lowest (below 25th percentile, TILs density-low) with those in the highest (above 75th percentile, TILs density-high) TILs density categories. Associations between groups and clinicopathological features including age (age ≤ 70 vs > 70 years), sex, tumour location (for early-stage trials only where pre-treatment tumour location information was available), pathological T stage ((y)pT) and lymph node status ((y)pN) were assessed using Mann–Whitney and Kruskal–Wallis tests, as appropriate. Relationship between TILs density and MMR status was investigated in MAGIC, CLASSIC and GO2 using continuous TILs density values. Patients with squamous cell carcinoma (SCC) or adenocarcinoma were included in OE02, COG and GO2 allowing to investigate the relationship between TILs density and clinicopathological variables stratified by histopathological subtype. Only for this subgroup analysis, the median TILs density was used as cut-off.* P*-values of < 0.05 were considered statistically significant. As this is an exploratory/hypothesis generating study, correction for multiple testing was not done.

## Results

In total, pre-treatment endoscopic biopsies from 2787 OG cancer patients and resection specimens from 3223 OG cancer patients across nine phase III clinical trials were available for H&E-based TILs density analysis. A total of 19,057 tumour regions were manually annotated by 13 investigators from the international UGI TILs working group (included as co-authors). This included 13,289 annotated endoscopic biopsy pieces and 5768 annotated resections*.* Basic characteristics of the clinical trials are summarised in Table 1. Table 2 provides details on tumour region sizes per specimen type and trial arm. For an illustration of the automatic TILs detection see Fig. 1.

**Table 2:** Size of tumour region used for TILs detection in patients included in the current study stratified by trial arm

Trial	Treatment arm	Pre-treatment biopsies				Resection specimens			
		n	Size of tumour region (mm^2^)			n	Size of tumour region (mm^2^)		
			median	min	max		median	min	max
OE02	Surgery alone	158	4.3	0.3	36.6	234	14.1	2.6	208.6
	CF + Surgery	146	3.8	0.04	54.0	224	14.0	1.1	125.6
OE05	CF + Surgery	384	5.3	0.1	56.9	344	14.1	3.9	41.5
	ECX + Surgery	373	5.6	0.1	51.5	298	14.0	1.7	36.6
MAGIC	Surgery alone	N/A				162	13.9	5.6	28.3
	ECF + Surgery + ECF					142	13.9	3.2	28.1
ST03	ECX + Surgery + ECX	429	5.0	0.04	30.8	380	14.0	0.4	56.5
	ECX + Bev + Surgery + ECX + Bev	420	4.4	0.03	59.2	347	14.0	0.6	54.5
REAL3	EOX	126	4.3	0.1	27.3	N/A			
	EOX + panitumumab	136	3.5	0.1	32.9				
COG	Placebo(0)	148	4.7	0.1	27.4	N/A			
	Gefitinib (1)	148	5.7	0.2	56.6				
GO-2	Dose level A	88	6.5	0.6	57.7	N/A			
	Dose level B	90	6.8	0.3	61.2				
	Dose level C	95	6.9	0.6	32.3				
	Best supportive care	7	8.3	2.6	13.3				
CLASSIC	Surgery alone	N/A				304	14.8	3.1	19.3
	Surgery + CAPOX					325	14.5	1.4	18.0
SAMIT	Surgery + S-1	N/A				125	14.1	7.1	52.1
	Surgery + UFT					126	14.1	8.4	103.5
	Surgery + paclitaxel, then UFT					126	14.1	6.1	102.9
	Surgery + paclitaxel, then S-1					125	14.1	8.1	52.1
Total		2787	5.0	0.0	61.2	3225	13.7	0.4	208.6

Abbreviations: CF: 5-fluorouracil + cisplatin. ECX: epirubicin + cisplatin + capecitabine. ECF: epirubicin + cisplatin + 5-fluorouracil. Bev: bevacizumab. EOX: epirubicin + oxaliplatin + capecitabine. CAPOX: capecitabine + oxaliplatin. UFT: tegafur + uracil. S-1: tegafur + gimeracil + oteracil. N/A = not applicable

### Tumour infiltrating lymphocytes per mm^2^ (TILs density) per trial, and geographical region

**Fig. 2:**
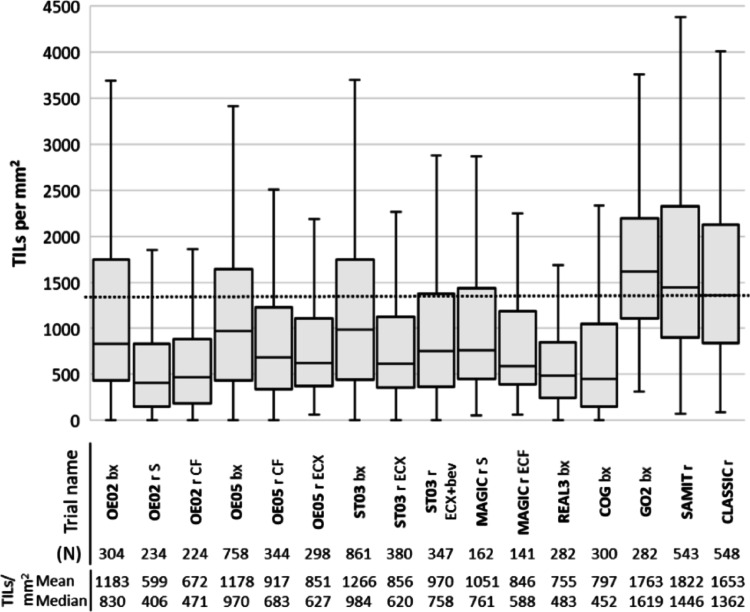
TILs density distribution across all trials. Box plot showing a comparison of the TILs density between individual trials, trial arms, disease group (early-stage vs late-stage) and specimen type (pre-treatment biopsies, resection specimens). Line within the box represents median. The dashed line represents median TILs density from the pilot study using the CLASSIC trial, to demonstrate that UK-led trials have lower median TILs densities. Abbreviations: bx = biopsy. r = resection. S = surgery alone. CF = cisplatin—5-fluorouracil. ECX = epirubicin – cisplatin – capecitabine. ECF = epirubicin – cisplatin – 5 -fluorouracil. Bev = bevacizumab

Figure 2 illustrates the TILs density across different trials by trial arm. The GO2 trial was excluded from this comparison due to its substantially higher median TILs density (1619 TILs/mm^2^), which made it an outlier amongst the UK late-stage disease trials. Table 3 presents TILs density stratified by geographical region, disease stage (early-stage vs late-stage disease) and specimen type (pre-treatment biopsies, pre-treatment resections, chemotherapy-naïve resections and post-chemotherapy resections). The median TILs density in the pre-treatment biopsies of patients with early-stage disease (OE02, OE05, ST03) was similar and significantly higher than in those with late-stage disease (REAL3 and COG): median (range) TILs density pre-treatment biopsies early-stage disease: 962 TILs/mm^2^ (1–11,219 TILs/mm^2^) versus late-stage disease: 479 TILs/mm^2^ (0–46,426 TILs/mm^2^), *p*-value < 0.001.

**Table 3:** TILs density (TILs/mm^2^) and interquartile ranges stratified by trial, specimen type, and different clinical trial groups

A. UK-led trials, resections, early stage disease										
	Surgery alone			Post-chemotherapy resection						
	OE02 S n = 221	MAGIC S n = 146	Combined (Group 1)	OE02 CF n = 225	OE05 CF n = 339	OE05 ECX n = 290	MAGIC ECF n = 124	ST03 ECX n = 353	ST03 ECX + Bev n = 325	Combined (Group 2)
Mean (± STD)	587 (642)	1030 (950)	763 (808)	683 (750)	913 (788)	843 (710)	815 (709)	825 (706)	943 (844)	849 (762)
Median	403	739	571	476	678	622	562	613	943	618
Minimum	0	52	0	0	4	64	0	2	2	0
Maximum	3762	7009	7009	6043	5039	3974	2948	4181	8493	8493
Low (P25)	153	451	223†	191	338	367	376	341	364	331†
High (P75)	835	1432	973†	900	1233	1086	1017	1113	1368	1152†

B. Asia-led trials, resections, and UK-led trials biopsies											
	Asia-led trials, resection, early stage disease			UK-led trials, pre-treatment biopsy, early stage disease				UK-led trials, pre-treatment biopsy, late stage disease			
	SAMIT n = 523	CLASSIC n = 548	Combined (Group 3)	OE02 n = 304	OE05 n = 755	ST03 n = 824	Combined (Group 4)	REAL3 n = 282	COG n = 291	GO-2 n = 280	Combined (Group 5)*
Mean (± STD)	1826 (1366)	1677 (1169)	1751 (1271)	1183 (1029)	1181 (1003)	1269 (1132)	1220 (1066)	755 (2770)	814 (968)	1761 (880)	758 (2061)
Median	1446	1408	1419	830	972	989	962	483	475	1761	479
Minimum	74	76	74	5	1	1	1	0	0	314	0
Maximum	9318	7989	9318	5767	5947	11,219	11,219	46,426	7164	5476	46,426
Low (P25)	914	863	888†	432	433	439	438†	244	166	1108	198†
High (P75)	2276	2124	2202†	1750	1646	1755	1698†	846	1062	2187	925†

Abbreviations: S: surgery alone. CF: 5-fluorouracil + cisplatin. ECX: epirubicin + cisplatin + capecitabine. ECF: epirubicin + cisplatin + 5- fluorouracil. Bev = bevacizumab. P25: 25th percentile. P75: 75th percentile. *Combined TILs density from biopsies of unresectable disease (Group 5) was calculated from REAL3 and COG, excluding GO-2. †: the P25 and P75 values for each group have been used for analysis of relationship with clinicopathological data in Table 4

Among UK-led trials in patients with early-stage disease, post-chemotherapy resections (OE02, OE05, ST03, MAGIC) had a significantly higher TILs density compared to chemotherapy-naïve resections (OE02, MAGIC): median (range) TILs density post-chemotherapy resections: 618 TILs/mm^2^ (0—8493 TILs/mm^2^) versus chemotherapy-naïve resections: 571 TILs/mm^2^ (0–7009 TILs/mm^2^), *p*-value = 0.003.

### Relationship between TILs density and geographical region of patient recruitment

The median (range) TILs density in the UK post-chemotherapy resections was similar across all trials (OE02, OE05, ST03, MAGIC), 618 TILs/mm^2^ (0—8493 TILs/mm^2^). The median (range) TILs density in the UK chemotherapy-naïve resection (OE02 and MAGIC) was significantly lower (571 TILs/mm^2^ (0—7009 TILs/mm^2^)) than in the Asian chemotherapy-naïve resections (1,419 TILs/mm^2^ (74–9,318 TILs/mm^2^)), *p*-value < 0.001.

### Relationship between TILs density and clinicopathological data

Given the substantial variability in TILs density across different disease settings, we grouped patients by disease setting (see Material and Methods, Fig. 2 and Table 3) and restricted the analysis to the comparison between TILs density-high and TILs density-low*.*

### Age

The median age of all patients from the OE02, OE05, ST03, REAL3, COG, MAGIC, SAMIT and CLASSIC trials was similar [63 years, range: 23–87 years] indicating a broadly comparable age distribution across these cohorts. In contrast, the median age of patients in the GO2 trial was 76 years due to the trial’s focus on an elderly/frail patient population. For pooled age analysis, patients were stratified into two categories: ≤ 70 years and > 70 years. GO2 trial patients were excluded from this analysis as only 25% (*n* = 69) patients in the GO2 trial were younger than 70 years. We found no significant relationship between TILs density and age per group (1–5) when comparing patients with TILs density-low tumours vs those with TILs density-high tumours, see Table 4. There was also no relationship between age and TILs density-low tumours versus TILs density-high tumours in the separately analysed GO2 trial patients.

**Table 4:** Relationship between low TILs density (below 25^th^ percentile) versus high TILs density (above 75^th^ percentile) and clinicopathological data in resection specimens (A) and biopsies (B) in different groups

A	UK-led trials, early stage disease								Asia-led trials, early stage disease			
	Resection								Group 3 (Pre-treatment resection)			
	Group 1 (Surgery alone)				Group 2 (Chemotherapy + surgery)							
	Total n = 367	TILs low (< 223 TILs/mm^2^)	TILs high (> 973 TILs/mm^2^)	p-value	Total n = 1656	TILs low (< 331 TILs/mm^2^)	TILs high (> 1152 TILs/mm^2^)	p-value	Total n = 1070	TILs low (< 888 TILs/mm^2^)	TILs high (> 2202 TILs/mm^2^)	p-value
		n (%)	n (%)			n (%)	n (%)			n (%)	n (%)	
*Age*												
≤ 70	295	77 (84)	69 (76)	0.243	1402	353 (85)	352 (85)	0.940	865	214 (80)	216 (81)	0.779
> 70	72	15 (16)	22 (24)		254	62 (15)	63 (15)		205	53 (20)	51 (19)	
*Sex*												
Female	96	28 (30)	20 (22)	0.075	270	77 (19)	70 (17)	0.313	314	80 (30)	77 (29)	0.742
Male	271	64 (70)	71 (78)		1386	338 (81)	345 (83)		756	187 (70)	190 (71)	
*Location*												
Oesophagus	57	25 (27)	7 (8)	** < 0.001**	47	15 (4)	10 (2)	0.213	0	0 (0)	0 (0)	0.536
Junction	198	56 (61)	46 (50)		1066	268 (68)	271 (68)		43	10 (4)	7 (3)	
Stomach	112	11 (12)	38 (42)		476	111 (28)	118 (30)		1027	257 (96)	260 (97)	
*(y)pT category*												
T1	30	8 (9)	12 (13.2)	0.334	163	21 (5)	62 (14.9)	** < 0.001**	11	1 (0)	2 (0.7)	**0.032**
T2	45	11 (12)	13 (14.3)		269	52 (12)	82 (19.8)		255	55 (21)	79 (29.6)	
T3	243	64 (69)	52 (57.1)		1014	268 (65)	235 (56.6)		240	137 (51)	130 (48.7)	
T4	49	9 (10)	14 (15.4)		210	74 (18)	36 (8.7)		264	74 (28)	56 (21)	
*(y)pN category*												
N0	112	38 (41)	29 (33)	0.141	574	121 (29)	160 (39)	**0.005**	152	33 (12)	47 (18)	0.055
N1 +	251	54 (59)	60 (67)		1076	290 (71)	254 (61)		917	234 (88)	220 (82)	

B	UK-led trials early and late stage disease							
	Group 4 (Pre-treatment biopsy early stage disease)				Group 5 (Pre-treatment biopsy late stage disease)			
	Total n = 1883	TILs low (< 438 TILs/mm^2^)	TILs high (> 1698 TILs/mm^2^)	p-value	Total n = 573	TILs low (< 198 TILs/mm^2^)	TILs high (> 925 TILs/mm^2^)	p-value
		n (%)	n (%)			n (%)	n (%)	
*Age*								
≤ 70	1576	393 (84)	397 (84)	0.472	423	103 (74)	105 (75)	0.671
> 70	307	77 (16)	73 (16)		130	37 (26)	35 (25)	
*Sex*								
Female	309	75 (16)	86 (18)	0.317	94	26 (19)	26 (19)	0.821
Male	1574	395 (84)	384 (82)		459	114 (81)	114 (81)	
*Location*								
Oesophagus	73	18 (4)	23 (5)	** < 0.001**				
Junction	1328	342 (74)	305 (65)					
Stomach	468	102 (22)	142 (30)					

### Sex

The proportion of male participants was higher in all trials, ranging from 64% in SAMIT to 90% in OE05. We found no significant relationship between TILs density and sex per group (1–5) when comparing patients with TILs density-low tumours vs those with TILs density-high tumours, see Table 4. There was also no relationship between sex and TILs density-low tumours versus TILs density-high tumours in the separately analysed GO2 trial patients.

### Tumour location

The relationship between TILs density and tumour location was analysed in the early-stage disease trials OE02, OE05, MAGIC, ST03, CLASSIC and SAMIT. In early-stage UK trials group 1 (surgery alone) 15% (n = 58) of patients had oesophageal cancer, 53% (n = 206) junctional cancer and 32% (n = 123) gastric cancer. There was a significant relationship between tumour location and TILs-density, *p*-value < 0.001 (see Table 4). However, no significant relationship between tumour location and TILs density was identified in group 2 (peri-operative chemotherapy) of the early-stage UK trials or in groups 3 (Asian-led trials). The relationship between tumour location and TILs density was not assessed in late-stage disease due to lack of tumour location data.

### DNA mismatch repair (MMR) status

The relationship between TILs density (continuous variable) and MMR status was analysed in MAGIC, CLASSIC and GO2 trials. The proportion of MMR-deficient tumours was 6.8% (n = 20) in MAGIC, 6.7% (n = 25) in CLASSIC, and 8.8% (n = 6) in GO2. There was no significant relationship between MMR status and TILs density across these studies. The OE02 trial was not included in this analysis due to the presence of only a single MMR-deficient tumour. MMR data were unavailable for the remaining trials.

### Pathological disease stage (depth of invasion ((y)pT) and lymph node status ((y)pN))

In the OE02, OE05, MAGIC, ST03 and SAMIT trials, the proportion of patients with (y)pT3 tumours ranged from 51% in MAGIC to 74% in OE02. In the CLASSIC trial, 45% of patients had pT4 tumours. Across all trials (excluding SAMIT and CLASSIC due to stage-based inclusion criteria), the majority of patients had lymph node metastasis ranging from 63% ypN1 + in ST03 to 70% ypN1 + in OE02 and MAGIC. We found a significant relationship with TILs density, (y)pT and (y)pN in group 2 and group 3 when comparing patients with TILs density-low tumours vs those with TILs density-high tumours. In group 2 (UK-led trials, post-chemotherapy resections), a higher ypT stage and a higher ypN stage was significantly associated with low TILs density, *p* < 0.001 and *p* = 0.005, respectively, see Table 4. In Group 3 (Asia-led trials, treatment-naïve resection), a higher pT stage was significantly associated with low TILs density, *p* = 0.032. There was no significant relationship between pN stage and TILs density (low vs high) in group 3 (Asian-led trials), see Table 4. However, a trend towards higher TILs density in patients with lower pN stage was noted (*p* = 0.055). This finding should be interpreted with caution due to the small number of pN0 patients included in the Asian trials.

### Subgroup analysis by histological subtype in OE02, COG, GO-2 trials

No significant differences in TIL density were observed between histological subtypes in biopsy specimens from the COG, GO-2, and OE02 trials. However, a significant difference was identified in OE02 resection specimens, where TIL density was lower in squamous cell carcinoma (SCC) compared with adenocarcinoma. The median (range) TIL density was 266 TILs/mm^2^ (0–3762) in SCC resections versus 503 TILs/mm^2^ (0–6043) in adenocarcinoma resections (*p* < 0.001).

Further analysis of clinicopathological variables by histological subtype demonstrated a significant relationship between TILs density and depth of invasion (ypT) in adenocarcinoma resection specimens in the OE02 trial. In post-chemotherapy resections, higher TILs density was associated with earlier ypT stage. In surgery-alone resections, higher TILs density was associated with more advanced pN stage. These associations were not observed in the SCC subgroup (see supplement Table 2A). There was no significant relationship between TILs density and age, sex or tumour location across histological subtypes (see supplement Tables 2A-C3).

## Discussion

This study is the first large-scale analysis of tumour infiltrating lymphocytes (TILs) density across nine phase III oesophago-gastric cancer clinical trials, providing new insights into the landscape of TILs and their heterogeneity across different clinical and demographic subgroups. We demonstrate that TILs density varies with disease stage and geographical region but not with age or sex. These findings highlight the need to consider different oesophagogastric (OG) cancer settings when defining clinically relevant TILs thresholds for patient stratification. Accordingly, we conclude that our previously published TILs density threshold from the Korean CLASSIC trial study [19] is not directly applicable in non-Asian OG cancer populations. Whilst this work lays the foundation for appropriate TILs density stratification, defining clinically meaningful thresholds for survival or response to therapy prediction was beyond the scope of the current study.

The higher TILs density in Asian patients in our study is consistent with results from breast and lung cancer [25, 26], and probably reflects differences in genetics, environment, and lifestyle, which may influence the host’s anti-tumour immunity. Such geographical differences suggest that there is a need for population-specific TILs density thresholds.

The higher TILs density observed in patients with gastric cancers, compared to junctional and oesophageal in the early-stage surgery alone group is consistent with prior literature describing a more immunologically active tumour microenvironment in gastric malignancies [53]. This relationship was not observed in patients who received neoadjuvant chemotherapy, suggesting that systemic treatment may modulate the tumour immune microenvironment. However, this finding should be interpreted with caution in the absence of additional immune-related biomarker, such as Epstein-Barr virus (EBV) infection status, which has been shown to be more frequent in gastric than in oesophageal cancer [52, 54].

The observed relationship between TILs density and disease stage is consistent with multi-cancer analyses showing reduced immune infiltration in more advanced cancers [36]. TILs density decreased with higher disease stage in both UK post-chemotherapy resections and Asian chemotherapy-naïve resections. This could suggest that early-stage tumours may elicit stronger immune surveillance preventing deeper invasion, while more advanced tumours may have greater immune evasion, reflected by reduced TILs infiltration.

Subgroup analyses by histological subtype (adenocarcinoma vs squamous cell carcinoma (SCC)) was possible only in few trials and revealed additional complexity. While there were no differences in TILs density in biopsies (OE02, COG, GO2) between adenocarcinoma and SCC, resection specimens from the OE02 trial showed lower TILs density in SCC. Furthermore, associations between TIL density and pathological stage were observed in adenocarcinoma but not in SCC, suggesting potential differences in immune–tumour interactions between histological subtypes. These findings warrant further investigation with a view to their potential relevance for immunotherapy selection in OG cancer.

No association between age and TILs density was observed in this study contrary to reports in other cancers [55, 56]. Notably, GO2 was excluded from the pooled analysis due its substantially higher level of TILs density compared to all other UK trials. GO2 exclusively enrolled older, frailer patients and the higher median TILs density in this cohort suggests that age-related differences in the tumour microenvironment may exist. The age threshold used in our analysis may not have captured biologically relevant differences in immune infiltration, and the lack of younger patients within GO2 limits direct assessment of age effects. Additionally, the narrow age ranges typical of the other clinical trial cohorts may further restrict generalisability. Consequently, the relationship between age and TILs density in oesophago-gastric cancer patients remains uncertain and warrants further investigation.

The current study has some limitations. This is a retrospective study using material from nine clinical trials originally designed to answer a clinical question rather than for TILs density evaluation. The variability in tissue processing across multiple centres may have introduced some bias. For example, significant staining differences in slides from the REAL3 and GO2 trials initially affected the performance of the deep learning model. These differences were addressed during manual quality control, and the DL model was further trained to reliably analyse these slides. From the resection specimens, areas with highest tumour density irrespective of their location within the tumour were sampled to maintain consistency with our previous study in the CLASSIC trial [19]. Furthermore, assessing TILs density in tumour-rich areas while avoiding immune cell dense regions may yield different results from measurements taken in immune hot spots. TILs were quantified on routine H&E stained tissue sections limiting insight into specific lymphocyte subpopulations and their relationship with clinicopathological features. However, quantification of TILs on routine H&E-stained tissue sections can be easily introduced into clinical practice as shown for TILs measurement in routine pathology specimens in breast cancer since 2018 [57]. Finally, unfortunately several important risk factors for carcinogenesis were not available for us to include in our analysis, such as Helicobacter pylori infection, Barrett’s oesophagus, and EBV status, which may impact TILs density. MMR status was only available in 4 trials, and the low frequency of MMR deficiency limits interpretation of these findings. Further analyses investigating the relationship of TILs density and survival or response to therapy are necessary to define prognostic and predictive cut-offs and to determine whether TILs assessment can guide personalised treatment and immunotherapy strategies in OG cancer.

In summary, this is the largest dataset to date assessing TILs in OG cancer from nine different randomised clinical trials reflecting diverse patient populations with matched mature and high quality clinicopathological data. We found significant variation of TILs densities across different disease settings including geographical region and tumour stage, with no association with age or sex. Importantly, we demonstrate that our previously in the Asian CLASSIC trial identified TILs density cut-off may not be applicable in non-Asian datasets. The consistency of TILs density post chemotherapy across different regimens suggests potential generalisability to other cytotoxic chemotherapy regimens such as FLOT [3]. The inclusion of historical trials such as OE02 and MAGIC facilitated the evaluation of TILs density in patients treated by surgery alone, providing valuable reference data.

## Supplementary Information

Below is the link to the electronic supplementary material.

Supplementary Material 1
