# Supplementary material for: The density of tumour infiltrating lymphocytes in oesophago-gastric cancer varies with disease stage, geographical region and treatment: a post hoc analysis of nine phase III clinical trials
Source: Gastric Cancer. 2026 May 5;29(4):762–76. doi: 10.1007/s10120-026-01739-6 (PMC13314682; doi:10.1007/s10120-026-01739-6)
Supplement: Supplementary file 1 — Supplementary Material 1 [file 10120_2026_1739_MOESM1_ESM.docx]

**Supplement Table 1:** TILs density (TILs/mm^2^) and interquartile ranges stratified by trial, specimen type, and histological subtype.

|  | **Resections** | | | | **Biopsies** | | | | | |
| --- | --- | --- | --- | --- | --- | --- | --- | --- | --- | --- |
| TILs density (TILs/mm^2^) | **Surgery alone** | | **Post-chemotherapy resection** | | **Early stage disease** | | **Late stage disease** | | | |
|  | **OE02** | **OE02** | **OE02** | **OE02** | **OE02** | **OE02** | **COG** | **COG** | **GO-2** | **GO-2** |
|  | **S** | **S** | **CF** | **CF** |  |  |  |  |  |  |
|  | **n=160** | **n=59** | **n=167** | **n=58** | **n=185** | **n=69** | **n=211** | **n=78** | **n=236** | **n=33** |
|  | **adeno** | **SCC** | **adeno** | **SCC** | **adeno** | **SCC** | **adeno** | **SCC** | **adeno** | **SCC** |
| Mean (±STD) | 652 (648) | 418 (609) | 727 (804) | 556 (557) | 1175 (918) | 1402 (1348) | 803 (924) | 840 (1086) | 1647 (860) | 1725 (871) |
| Median | 512 | 230 | 499 | 414 | 888 | 871 | 457 | 840 | 1647 | 1539 |
| Minimum | 1 | 0 | 0 | 4 | 5 | 5 | 0 | 0 | 314 | 405 |
| Maximum | 3170 | 3762 | 6043 | 2429 | 4837 | 5767 | 5594 | 7164 | 5023 | 3428 |
| Low (P25) | 177 | 42 | 217 | 127 | 476 | 366 | 176 | 153 | 1108 | 1109 |
| High (P75) | 880 | 602 | 977 | 817 | 1762 | 2151 | 1049 | 1078 | 2170 | 2428 |

Abbreviations: S: surgery alone. CF: 5-fluorouracil+ cisplatin. P25: 25^th^ percentile. P75: 75^th^ percentile. Adeno: adenocarcinoma. SCC: squamous cell carcinoma.

**Supplement Table 2:** Relationship between low TILs density (below median) versus high TILs density (above median) and clinicopathological data stratified by histological subtypes in OE02, COG and GO2 trials.

| A. | **OE02 Surgery alone resections** | | | | | | | |
| --- | --- | --- | --- | --- | --- | --- | --- | --- |
|  | adenocarcinoma | | | | squamous cell carcinoma | | | |
|  | Total | TILs low (≤512 TILs/mm^2^) | TILs high (>512 TILs/mm^2^) | p-value | Total | TILs low (≤230 TILs/mm2) | TILs high (>230 TILs/mm^2^) | p-value |
|  | n = 160 | n (%) | n (%) |  | n = 59 | n (%) | n (%) |  |
| **Age** | | | | | | | | |
| ≤70 | 126 | 64 (80) | 62 (77.5) | 0.428 | 48 | 25 (83) | 23 (79) | 0.586 |
| >70 | 34 | 16 (20) | 18 (22.5) |  | 11 | 5 (17) | 6 (21) |  |
| **Sex** | | | | | | | | |
| Female | 26 | 17 (21) | 9 (11) | 0.127 | 31 | 17 (57) | 14 (48) | 0.952 |
| Male | 134 | 63 (79) | 71 (89) |  | 28 | 13 (43) | 15 (52) |  |
| **Location** | | | | | | | | |
| Oesophagus | 20 | 11 (14) | 9 (11) | 0.483 | 37 | 19 (63) | 18 (62) | 0.510 |
| Junction | 140 | 69 (86) | 71 (89) |  | 22 | 11 (37) | 11 (38) |  |
| **(y)pT category** | | | | | | | | |
| T1 | 11 | 6 (7.5) | 5 (6) | 0.296 | 7 | 2 (7) | 5 (17) | 0.173 |
| T2 | 20 | 13 (16) | 7 (9) |  | 3 | 2 (7) | 1 (3) |  |
| T3 | 120 | 55 (69) | 65 (81) |  | 48 | 25 (83) | 23 (79) |  |
| T4 | 9 | 6 (7.5) | 3 (4) |  | 1 | 1 (3) | 0 (0) |  |
| **(y)pN category** | | | | | | | | |
| N0 | 35 | 22 (27.5) | 13 (16) | **0.007** | 33 | 16 (53) | 17 (59) | 0.831 |
| N1+ | 125 | 58 (72.5) | 67 (84) |  | 26 | 14 (47) | 12 (41) |  |

| B. | **OE02 Post-chemotherapy resection** | | | | | | | |
| --- | --- | --- | --- | --- | --- | --- | --- | --- |
|  | adenocarcinoma | | | | squamous cell carcinoma | | | |
|  | Total | TILs low (≤499 TILs/mm2) | TILs high (>499 TILs/mm^2^) | p-value | Total | TILs low (≤414 TILs/mm2) | TILs high (>414 TILs/mm^2^) | p-value |
|  | n = 167 | n (%) | n (%) |  | n = 58 | n (%) | n (%) |  |
| **Age** | | | | | | | | |
| ≤70 | 135 | 66 (80) | 69 (82) | 0.518 | 43 | 24 (83) | 19 (66) | 0.138 |
| >70 | 32 | 17 (20) | 15 (18) |  | 15 | 5 (17) | 10 (34) |  |
| **Sex** | | | | | | | | |
| Female | 19 | 11 (13) | 8 (10) | 0.751 | 26 | 15 (52) | 11 (38) | 0.364 |
| Male | 148 | 72 (87) | 76 (90) |  | 32 | 14 (48) | 18 (62) |  |
| **Location** | | | | | | | | |
| Oesophagus | 16 | 7 (8) | 9 (11) | 0.378 | 31 | 13 (45) | 18 (62) | 0.246 |
| Junction | 151 | 76 (92) | 75 (89) |  | 27 | 16 (55) | 11 (38) |  |
| **(y)pT category** | | | | | | | | |
| T1 | 16 | 8 (9.6) | 8 (10) | **0.014** | 3 | 0 (0) | 3 (10) | 0.469 |
| T2 | 19 | 4 (4.8) | 15 (18) |  | 13 | 7 (24) | 6 (21) |  |
| T3 | 123 | 63 (75.9) | 60 (71) |  | 42 | 22 (76) | 20 (69) |  |
| T4 | 9 | 8 (9.6) | 1 (1) |  | 0 | 0 | 0 |  |
| **(y)pN category** | | | | | | | | |
| N0 | 59 | 27 (33) | 32 (38) | 0.300 | 32 | 15 (52) | 17 (59) | 0.755 |
| N1+ | 108 | 56 (67) | 52 (92) |  | 26 | 14 (48) | 12 (41) |  |

| C1. | **OE02 Biopsies** | | | | | | | |
| --- | --- | --- | --- | --- | --- | --- | --- | --- |
|  | adenocarcinoma | | | | squamous cell carcinoma | | | |
|  | Total | TILs low (≤830 TILs/mm2) | TILs high (>830 TILs/mm^2^) | p-value | Total | TILs low (≤830 TILs/mm2) | TILs high (>830 TILs/mm^2^) | p-value |
|  | n = 185 | n (%) | n (%) |  | n = 69 | n (%) | n (%) |  |
| **Age** | | | | | | | | |
| ≤70 | 146 | 63 (72) | 83 (85) | 0.241 | 56 | 25 (76) | 31(86) | 0.182 |
| >70 | 39 | 24 (28) | 15 (15) |  | 13 | 8 (24) | 5 (14) |  |
| **Sex** | | | | | | | | |
| Female | 26 | 11 (13) | 15 (15) | 0.788 | 31 | 14 (42) | 17 (47) | 0.856 |
| Male | 159 | 76 (87) | 83 (85) |  | 38 | 19 (58) | 19 (53) |  |

| C2. | **COG Biopsies** | | | | | | | |
| --- | --- | --- | --- | --- | --- | --- | --- | --- |
|  | adenocarcinoma | | | | squamous cell carcinoma | | | |
|  | Total | TILs low (≤475 TILs/mm2) | TILs high (>475 TILs/mm^2^) | p-value | Total | TILs low (≤475 TILs/mm2) | TILs high (>475 TILs/mm^2^) | p-value |
|  | n = 211 | n (%) | n (%) |  | n = 78 | n (%) | n (%) |  |
| **Age** | | | | | | | | |
| ≤70 | 149 | 74 (69) | 75 (72) | 0.735 | 56 | 25 (66) | 31 (77.5) | 0.368 |
| >70 | 62 | 33 (31) | 29 (28) |  | 22 | 13 (34) | 9 (22.5) |  |
| **Sex** | | | | | | | | |
| Female | 25 | 13 (12) | 12 (12) | 0.936 | 25 | 13 (34) | 12 (30) | 0.712 |
| Male | 186 | 94 (88) | 92 (88) |  | 53 | 25 (66) | 28 (70) |  |

| C3. | **GO2 Biopsies** | | | | | | | |
| --- | --- | --- | --- | --- | --- | --- | --- | --- |
|  | adenocarcinoma | | | | squamous cell carcinoma | | | |
|  | Total | TILs low (≤1617 TILs/mm2) | TILs high (>1617 TILs/mm^2^) | p-value | Total | TILs low (≤1617 TILs/mm2) | TILs high (>1617 TILs/mm^2^) | p-value |
|  | n = 236 | n (%) | n (%) |  | n = 33 | n (%) | n (%) |  |
| **Age** | | | | | | | | |
| ≤70 | 59 | 26 (23) | 33 (27) | 0.683 | 7 | 4 (22) | 3 (20) | 0.826 |
| >70 | 177 | 88(77) | 89 (73) |  | 26 | 14 (78) | 12 (80) |  |
| **Sex** | | | | | | | | |
| Female | 45 | 23 (20) | 22 (18) | 0.962 | 15 | 6 (33) | 9 (60) | 0.148 |
| Male | 191 | 91 (80) | 100 (92) |  | 18 | 12 (67) | 6 (40) |  |
